# Genome Sequence of the Banana Pathogen *Dickeya zeae* Strain MS1, Which Causes Bacterial Soft Rot

**DOI:** 10.1128/genomeA.00317-13

**Published:** 2013-06-13

**Authors:** Jing-Xin Zhang, Bi-Run Lin, Hui-Fang Shen, Xiao-Ming Pu

**Affiliations:** Key Laboratory of New Technique for Plant Protection in Guangdong, Institute of Plant Protection, Guangdong Academy of Agricultural Sciences, Guangdong, China

## Abstract

We report a draft genome sequence of *Dickeya zeae* strain MS1, which is the causative agent of banana soft rot in China, and we show several of its specific properties compared with those of other *D. zeae* strains. Genome sequencing provides a tool for understanding the genomic determination of the pathogenicity and phylogeny placement of this pathogen.

## GENOME ANNOUNCEMENT

*Dickeya zeae* MS1 is the causative agent of soft rot disease in bananas. It was first found in China by Lin et al. in 2009 (1) and has since become a major pathogen affecting the banana industry. The disease is characterized by massive odorous soft rot at the center or a portion of the rhizome, deteriorated with a destroyed growing point and decayed internal material, leading to vascular discoloration. Moreover, the organism shows a specific gray thallus in culture compared with other *Dickeya* pathogens, and it is short of the phytotoxin against a range of bacterial pathogens compared with other *D. zeae* strains (2, 3).

To gain further insights into the genomic determination of its pathogenicity and specific properties, the draft genome of MS1 was sequenced and annotated. Genomic DNA was isolated with the TIANamp bacterial DNA kit (Tiangen Biotech, Beijing, China), and genomic sequencing using the Roche GS FLX Titanium platform was performed at Macrogen. The shotgun library yielded 777,832 reads with an average length of 458 bp, amounting to 356,558,919 bases (approximately 75.09× coverage). The proportion of Q40+ bases was 99.99%, and high-quality reads were assembled in Newbler assembler v2.6 (454), yielding 58 contigs (>500 bp). Automated gene findings were made with Glimmer (4), and functional annotation was performed by means of similarity searches against the GO (5), COGs (6) and KEGG (7) databases.

The draft genome of strain MS1 is 4,748,283 bp in length with 53.3% G+C content, and further inspection found 4,465 predicted protein-coding genes. Functional annotation revealed virulence-related genes, including type III and type VI secretion system gene clusters, and pectinase, twin-arginine translocation (Tat) protein, and flagellins involved in the pathogenicity mechanisms of *Enterobacteriaceae*. In another case, the multiple antibiotic resistance (MarC)-related protein, the multidrug resistance protein MdtG, drug resistance transporters, and several kinds of efflux pumps were identified in the MS1 genome. Additionally, 42 genes were found to be involved in the sulfur metabolism pathway and might be related to the fact that the pathogen prefers to grow under anaerobic conditions (8).

Whole-genome sequencing was carried out not only to determine the genetic basis of characteristics and virulence-related genes, but also to compare genome data with those of the already published genomes of *D. zeae* Ech1591, *Dickeya dadantii* 3937, *D. dadantii* Ech586, and *D. dadantii* Ech703, contributing to the study of its phylogeny placement, taxonomy, and genomic differentiation. Of the predicted genes, 314 predicted functional genes are unique to reference strain *D. zeae* Ech1591, and 82 genes were not found in other *Dickeya* genomes, including 22 homologous genes in the *Pectobacterium* genus. Further, five clustered regularly interspaced short palindromic repeat (CRISPR) arrays providing acquired immunity against external invasion (9) were detected in the MS1 genome using CRISPRFinder (10) and varied with those of four CRISPR arrays found in *D. zeae* Ech1591, *D. dadantii* 3937, and *D. dadantii* Ech703 and six in *D. dadantii* Ech586.

### Nucleotide sequence accession numbers.

This Whole-Genome Shotgun project has been deposited at DDBJ/EMBL/GenBank under the accession no. APWM00000000. The version described in this paper is the first version, accession no. APWM01000000.
